# Analysis of Fixed and Live Single Cells Using Optical Photothermal Infrared with Concomitant Raman Spectroscopy

**DOI:** 10.1021/acs.analchem.0c04846

**Published:** 2021-02-17

**Authors:** Alice Spadea, Joanna Denbigh, M. Jayne Lawrence, Mustafa Kansiz, Peter Gardner

**Affiliations:** † NorthWest Centre for Advanced Drug Delivery (NoWCADD), School of Health Sciences, University of Manchester, Oxford Road, Manchester M13 9PL, U.K.; ‡ Division of Pharmacy and Optometry, Faculty of Biology, Medicine and Health, University of Manchester, Manchester Academic Health Science Centre Oxford Road, Manchester M13 9PL, U.K.; § Seda Pharmaceutical Development Services, Alderley Park, Alderley Edge, Cheshire SK10 4TG, U.K.; ∥ School of Science, Engineering and Environment, University of Salford, Salford, M5 4WT, U.K.; ⊥ Photothermal Spectroscopy Corp. 325 Chapala Street, Santa Barbara, California 93101, United States; # Manchester Institute of Biotechnology, University of Manchester, 131 Princess Street, Manchester M1 7DN, U.K.; ¶ Department of Chemical Engineering and Analytical Science, School of Engineering, University of Manchester, Oxford Road, Manchester M13 9PL, U.K.

## Abstract

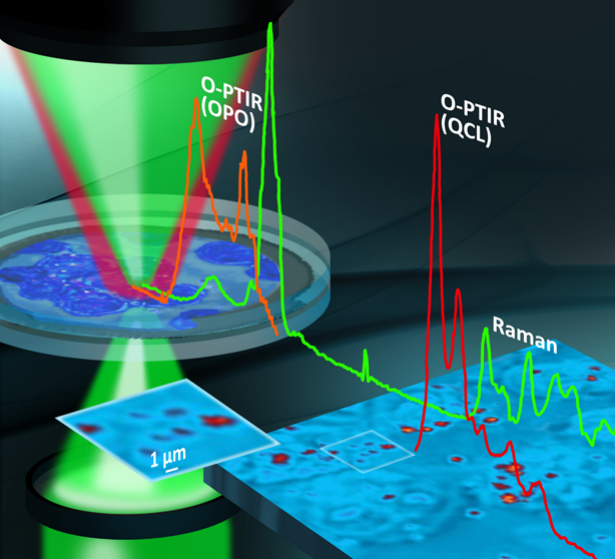

This paper reports the first use of a novel completely optically based photothermal method (O-PTIR) for obtaining infrared spectra of both fixed and living cells using a quantum cascade laser (QCL) and optical parametric oscillator (OPO) laser as excitation sources, thus enabling all biologically relevant vibrations to be analyzed at submicron spatial resolution. In addition, infrared data acquisition is combined with concomitant Raman spectra from exactly the same excitation location, meaning the full vibrational profile of the cell can be obtained. The pancreatic cancer cell line MIA PaCa-2 and the breast cancer cell line MDA-MB-231 are used as model cells to demonstrate the capabilities of the new instrumentation. These combined modalities can be used to analyze subcellular structures in both fixed and, more importantly, live cells under aqueous conditions. We show that the protein secondary structure and lipid-rich bodies can be identified on the submicron scale.

Vibrational spectroscopy is being increasingly used for the analysis of biological cells, tissues, and fluids. Both infrared and Raman spectroscopy provide complementary information relating to structure, function, and biochemical changes associated with a disease state or as a result of intentional exposure to stimuli.^1−4^ This interest is stimulated by an urgent need to develop label-free methods for chemically specific imaging while obtaining global biochemical information.

Fourier transform infrared (FTIR) imaging goes some way to filling this need.^5−7^ There are, however, a number of limitations with this methodology. Spatial resolution is typically the most important instrument parameter for microscopy, but for traditional infrared microscopy, the spatial resolution is significantly worse when compared to optical microscopy. Infrared wavelengths (λ) normally used are approximately 2.5–12.5 μm (4000–800 cm^–1^), meaning that, for an optimized nonconfocal microscope, the spatial resolution is limited to at best λ, making subcellular structures difficult to identify and resolve in all but the larger cell types.^8,9^ Using microscope objectives with larger numerical apertures (>0.6 NA) and substantial oversampling can improve the fidelity of the images, but the resolution is still limited to several micrometers essentially matching the Rayleigh criterion given by^10−14^
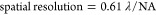
1Discrete frequency imaging using quantum cascade lasers (QCLs) has a significant advantage in speed, but the advantage in resolution in the wide field is still limited by the longer wavelength of the IR beam.^15−19^

The use of attenuated total reflection (ATR) as a measurement modality with FTIR microscopy can increase spatial resolution due to the high refractive index (RI) of the ATR crystal.^20^ This methodology does have limitations in that the sample must be prepared on the ATR crystal or the ATR crystal needs to make contact with the sample and as such, this significantly limits the throughput of the experiment^21^ and introduces potential for cross-contamination, sample damage, and/or ATR crystal damage. Near-field techniques, such as atomic force microscopy-infrared (AFM-IR) and scanning near-field optical microscopy (SNOM), can achieve higher spatial resolution, but the requirement to have a mechanical AFM tip in contact with or, very close to, the samples means that live cell imaging in an aqueous environment is extremely challenging^22−25^ and, therefore, is not widely employed. A new approach using closed-loop control to maintain zero amplitude cantilever deflection may substantially improve the situation, but this has yet to be demonstrated on single cells.^26,27^

Challenges associated with single-cell spectroscopy extend beyond the instrumentation. The vast majority of infrared single-cell analyses to date have reported the use of chemically or cryogenically fixed, dried cells. These preservation methods allow the biochemistry to be captured at a specific moment in time and have enabled the elucidation of significant information on cell behavior, including insights into disease progression and response to treatment across a wide range of cell types.^28−37^ The fixation of cellular samples and their presentation in dried form, however, introduces a range of spectral artifacts, most notably dispersive artifacts such as Mie and resonant Mie scattering.^38,39^ These are most prominent at sample RI discontinuities, such as sample-air gaps and edges, which lead to potential severe spectral distortions. While there are algorithms available to attempt to correct for some of these,^40−46^ often such corrections are not entirely accurate in reproducing a nondistorted spectrum. Reference spectra are required, and algorithms are computationally intensive; hence, it is preferable to eliminate these experimentally using some form of RI matching.

Live cell imaging helps to reduce these spectral artifacts due to there being little RI differences within and across samples.^31,47−50^ The aqueous environment required, however, introduces a significant additional challenge.^51^ Water is a strong IR absorber, meaning that live cell imaging in an aqueous environment is extremely difficult since the features in the cell spectrum, particularly the amide I vibration associated with proteins, can be obscured by the strong and broad bending mode of water centered at ∼1630–1640 cm^–1^.^52,53^ One way to address this problem is to use a high-brightness IR source. A number of papers have employed synchrotron FTIR, but the experiments are far from trivial and involve significant compromise in the spectral information obtained.^41,47,54,55^

Recently, however, a completely optically based photothermal method of obtaining infrared spectra has been developed that overcomes some of the limitations of FTIR.^56^ Based on the well-established principles of thermal lens spectroscopy^57^ and termed optical photothermal infrared (O-PTIR) spectroscopy, this technique is essentially a pump–probe setup, whereby the pump is the pulsed IR laser and the probe is a visible (green, 532 nm) laser. When the IR laser is focused onto the sample and tuned across its wavelength range, at wavelengths that correspond to a vibrational mode of the sample, local modulated sample heating occurs, causing subtle modulated sample expansion and RI changes as the IR energy is absorbed. This manifestation of IR absorption is termed the “photothermal infrared” effect.^57−63^

To detect this photothermal infrared effect, the visible probe is colinearly focused, together with the pump IR beam. The intensity of the reflected or transmitted (optical mode-dependent) probe beam is monitored as a function of IR wavelength using a high-sensitivity room-temperate silicon photodiode. Any modulated changes, at the same IR pulse rate, are demodulated, and pure IR spectra are extracted. Most importantly, the spatial resolution obtained is now determined by the shorter wavelength visible beam, not the IR beam and so is significantly better (up to 30×) than conventional FTIR microscopy. Further details of the optical system can be found in Figure S1. Previous O-PTIR studies have used tunable QCLs as the infrared pump beam. This enables spectra to be obtained in the region ∼1900–900 cm^–1^;^64^ however, the important region containing the C–H stretching vibrations is not captured.

In this paper, we present data obtained using both the QCL and a tunable optical parametric oscillator (OPO) laser that for the first time enables the higher wavenumber region (3600–2700 cm^–1^) containing the O–H, N–H, and C–H stretching vibrations to be accessed by O-PTIR. Additionally, the Raman spectrum from the visible probe beam can also be detected, enabling O-PTIR and concomitant Raman spectra to be recorded simultaneously from the exact same location, at the same time and with the same spatial resolution for both QCL and OPO laser excitations. Importantly, the two spectroscopic methods are complementary, meaning that vibrational signatures that are strong in one are often weak in the other and vice versa. This means that with both types of spectra obtainable from the same cellular location, a full vibrational spectroscopic profile can be obtained without the need for complex image registration.

Here, we demonstrate that the new combination of these modalities can be used to analyze subcellular structure in both fixed and, more importantly, live cells under aqueous conditions. In this study, the pancreatic cancer cell line MIA PaCa-2 and the breast cancer cell line MDA-MB-231 are used as model cells.

## Materials and Methods

### Chemicals and Reagents

Phosphate-buffered saline (PBS) tablets were purchased from Oxoid (Hampshire, U.K.). Cell culture media, l-glutamine, and a solution of 0.25% w/v trypsin and 0.02% w/v ethylenediaminetetraacetic acid (EDTA) were obtained from Sigma-Aldrich (Dorset, U.K.). Heat-inactivated fetal bovine serum (FBS) was supplied by GIBCO (Paisley, U.K.), untreated FBS was obtained from HyClone, Cytiva (Buckinghamshire, U.K.), and dimethyl sulfoxide (DMSO) was obtained from Fisher (Leicestershire, U.K.). Petri dishes, plates, and flasks for routine tissue culture were obtained from Falcon (Cheshire, U.K.). CaF_2_ (13 mm diameter x 1 mm thick) windows were supplied by Crystran (Dorset, U.K.). Unless otherwise stated, all other reagents and chemicals were supplied by Sigma-Aldrich (Dorset, U.K.).

### Instrumentation

A schematic of the optical layout of the O-PTIR system is shown in Figure S1. O-PTIR and concomitant Raman spectroscopy were carried out using a mIRage IR microscope (Photothermal Spectroscopy Corporation, Santa Barbara, CA) integrated with a four-module-pulsed quantum cascade laser (QCL) system, with a tunable range from 1890 to 790 cm^–1^. The IR laser repetition rate was set at ca. 110 kHz at 300 ns per pulse. The system is also equipped with a tunable OPO-pulsed infrared laser to cover the higher wavenumber region, with a tunable range from 3593 to 2693 cm^–1^. The system is coupled to a Horiba Scientific iHR-320 Imaging spectrometer that we have repurposed and fitted to the mIRage system. This has a 100 μm slit width and a grating of 600, 1200, and 1800 l/mm, all blazed at 500 nm. The key difference between this and the commercial variant available from Photothermal at the time is that the charge-coupled device (CCD) camera is front-illuminated with a pixel size of 26, whereas the commercial system was back-illuminated but with a 14 μm pixel size. The larger pixel size resulted in approximately a factor of 2 improvement in the signal-to-noise ratio of the Raman spectra.

### Cell Culture

MIA PaCa-2 and MDA-MB-231 cell lines (pancreatic and breast cancer, respectively) were grown in a humidified atmosphere at 37 °C, 5% CO_2_ (v/v), in T75 flasks with complete Dulbecco’s modified Eagle’s medium (DMEM) media. Prior to the spectroscopic measurements, the harvested cells were seeded at a density of ∼25 000 cells/cm^2^ onto sterile 0.5 mm thick, 25 mm diameter calcium fluoride (CaF_2_) windows placed in a six-well plate. The cells were left to attach overnight and then either fixed with 10% (w/v) formalin solution (3.7% formaldehyde) for 10 min or used live for the spectroscopic experiments.

### Confocal Microscopy Analysis

MIA PaCa-2 cells were seeded at a density of ∼25 000 cells/well in an 8-well μ-Slide (Ibidi, U.K.) as previously reported.^65^ Hoechst and Lysotracker (Invitrogen, U.K.) or Calnexin and GM130 antibodies (Santa Cruz, U.K.) were used to highlight, respectively, nuclei, lysosomes, endoplasmic reticulum, and Golgi. Briefly, cells were incubated with 0.3 mL of the 1 μg/mL solution of Hoechst in cell culture media for 10 min at 37 °C, 5% (v/v) CO_2_. To study the lysosomes, cells were stained live and a 1:5000 dilution of Lysotracker in cell culture media was added to the cells and incubated for 10 min under the same conditions. Cells were then washed, and media without phenol red was added and immediately used for confocal image assessment. To study endoplasmic reticulum structures, after Hoechst staining, cells were fixed with 10% (w/v) formalin for 10 min at room temperature. Prior to staining, cells were permeabilized with a 0.1% v/v TritonX solution in PBS incubating at room temperature (RT) for 2 min. Cells were then blocked with 1% (w/v) bovine serum albumin (BSA) and 10% (v/v) FBS in PBS for 30 min at RT. Calnexin and GM130 antibodies were made up to 1:100 in a blocking buffer. A volume of 100 μL of diluted antibodies was added to the cells and incubated for 1 h at 37 °C. The fluorochrome-conjugated secondary antibody was made up to 1:1000 in 1% BSA (v/v) in PBS and 100 μL was added to the cells and incubated for 1 h at 37 °C. Confocal acquisitions were performed with a Leica TCS SP5 AOBS inverted confocal using an oil 63×/0.60–1.40/HCX PL Apo objective and a 4× confocal zoom. The confocal settings were as follows: pinhole 1 airy unit, scan speed 400 Hz unidirectional, format 1024 × 1024. To eliminate any possible crosstalk between channels, images were collected with a sequential scan using the following laser lines and mirror settings: 405 (100%) nm, 410–483 nm; 488 (25%) nm, 495–550 nm; 561 (100%) nm. Images were then processed and analyzed using ImageJ 1.48v software (http://rsb.info.nih.gov/ij).

### Development of the Protocol for Analysis of Live Cells

For experiments, the windows with the live cells attached were washed three times with PBS and then using a peroxidase–antiperoxidase (PAP) pen (Sigma-Aldrich, U.K.), a thin hydrophobic film was deposited on the edge of the window, as shown in the scheme in Figure S2. A similar procedure has been previously tested and adopted by ourselves.^42^ A clean (cell-free) CaF_2_ window was carefully placed on top and pressed down until a hermetically sealed “sandwich” was obtained: the border drawn with the PAP pen prevented leakage of the buffer, thereby ensuring the cells were hydrated for up to 12 h (longer times were not tested). Once prepared, the sandwich was placed into a sample holder upside down, such that the cells were on the top CaF_2_ window used for analysis with the O-PTIR microscope.

### Spectra Acquisition and Data Processing

The optical images were acquired using the low-magnification 10× refractive objective, with a working distance of 15 mm and with the high-magnification (IR/visible) 40×, 0.78 NA (8 mm working distance) all-reflective Cassegrain objective. The O-PTIR spectra and images were collected with the same 40× objective. Wavenumber was calibrated against a polystyrene standard at 1601 ± 1 cm^–1^, and spectra were collected and processed using PTIRStudio 4.1. The hyperspectral map size for the fixed cells was 37 μm × 27.4 μm and a step size of 1 μm. The laser powers of the QCL IR Pump and green (532 nm) probe laser were set to 100 and 50%, respectively. O-PTIR spectra were collected with an effective spectral resolution of ca. 2 cm^–1^ and coaveraged for two spectral scans. The laser pulse rate was 100 kHz with a duty cycle of 2%, a pulse width of 200 nm, and a gain of 1×. For the OPO, the images were obtained using an IR power of 100%, a pulse rate of 86 kHz, a duty cycle of 1.72%, a pulse width of 200 nm, spectral averages of 2, a probe power of 50%, a gain of 1×, and a spectral resolution of 4 cm^–1^. The concomitant Raman spectra were collected using a Horiba Scientific iHR-320 Imaging Spectrometer with a focal length of 320 mm and f/4.1 aperture, with a choice of three diffraction gratings of which the 600 line/mm was used to give an approximate average spectral resolution of 6 cm^–1^. The grating position was centered to 2500 cm^–1^ giving a spectral range of 4029–561 cm^–1^. Spectra were obtained using spectral averages of 10, a probe power of 50%, and an integration time of 3 s. A thermoelectrically cooled Horiba Syncerity charged coupled device (CCD) detector was coupled to the spectrometer. The point spectra for the live cells with QCL laser were obtained at an IR power of 100%, a pulse rate of 100 kHz, a duty cycle of 2%, a pulse width of 200 nm, spectral averages of 2, a probe power of 25%, a gain of 1×, and a spectral resolution of 2 cm^–1^. Detector: transmission. For the OPO laser, the IR power was 100%, pulse rate 86 kHz, duty cycle 1.72%, spectral averages 2, probe power 50%, gain 1×, and spectral resolution 4 cm^–1^. The concomitant Raman spectra were obtained using spectral averages of 5, a probe power of 25%, and an integration time of 10 s. IR and Raman spectra underwent a Savitsky–Golay smoothing function, third-order polynomial, with eight side points.

## Results

### O-PTIR and Concomitant Raman for Subcellular Imaging of Fixed Cells

#### Overview

**Figure 1:**
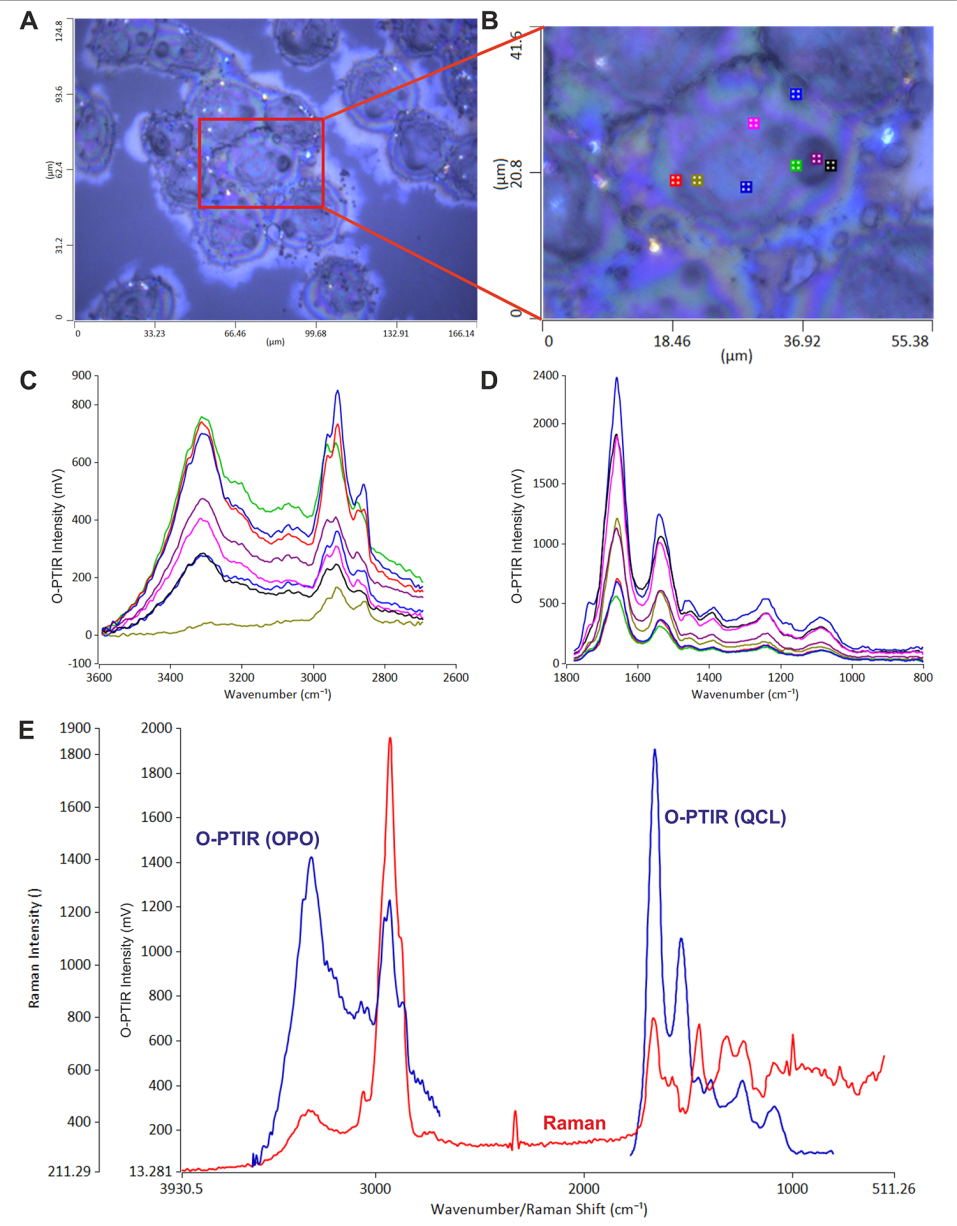
(A) Optical image of fixed unstained MIA PaCa-2 cells with the central cell shown expanded in (B). (C) Representative O-PTIR spectra, not normalized, from selected locations within the cell indicated by the colored spots in (B) obtained using OPO laser excitation covering the range 3593–2693 cm^–1^. (D) Representative O-PTIR spectra, not normalized, obtained using QCL laser excitation covering the range 1800–800 cm^–1^. (E) Concomitant Raman spectra (red lines) obtained from the black spot in panel (B) covering the range 3930–511 cm^–1^, shown with the representative O-PTIR spectra (blue lines).

Figure 1A shows an optical image of a cluster of unstained pancreatic MIA PaCa-2 cells. The central cell in the image is shown expanded in Figure 1B along with the locations from where representative infrared spectra have been obtained. Figure 1C shows the O-PTIR spectra, covering the range 3593–2693 cm^–1^, obtained using the OPO laser at selected locations from the cell, which are indicated by the corresponding marker colors in Figure 1B. The spectra clearly show the expected peaks associated with proteins and lipids within the cell (see Table S1), with the main variation in intensity due to the differing thickness across the cell.^66^ A recent study on polystyrene beads has shown that the O-PTIR signal is broadly related to the amount of material being sampled,^67^ but for a complex biological cell, it is possible that deviations from the Beer–Lambert law could occur.

Detailed analysis shows that there are also more noticeable spectral variations related to the location within the cell, which are due to the inherent biochemical heterogeneity. In the high wavenumber region, the first two main features are the large broad amide A band at ∼3300 cm^–1^ and the much smaller amide B feature at ∼3060 cm^–1^. The intensities of these bands are quite different depending upon the location within the cell but shift very little in band shape or position. This is not too surprising since, unlike the amide I band discussed later, these protein features are less sensitive to the molecular environment. Figure 1D shows the spectra obtained for the lower wavenumber region of 1800–800 cm^–1^ using QCL excitation at the locations indicated in Figure 1B. Again, there are variations in the intensity of the overall spectral profile reflecting mainly different thicknesses of the cell at specific locations. The two largest peaks in this region, namely, the amide I and II at ∼1656 and 1550 cm^–1^, respectively, reflect the protein content of the cell. Although collected in two parts, the O-PTIR spectra cover the entire biologically significant range (the region between these ranges is often termed biologically silent since it contains nonbiologically relevant information^68^) and agree very well with that expected based on conventional FTIR spectra of fixed cells (see Table S1(29−37)). Figure 1E shows the full Raman spectrum obtained concomitantly with the QCL data acquisition. OPO data, from the same spot, are also shown in the figure. The Raman spectrum covers the full spectral range, and immediately obvious is the complementarity of the two techniques. This is illustrated dramatically by the reduction of the vibrations associated with highly polar groups, i.e., N–H stretch (∼3300 cm^–1^) and amide I (1656 cm^–1^) in the Raman spectrum compared with that of the O-PTIR, but the increase in bands associated with the nonpolar groups, mainly the C–H vibrations (∼3050–2800, ∼1446, and ∼1330 cm^–1^). This demonstrates the advantage of being able to have both spectral modalities capable of acquiring data from the same spot at the same time.

#### Analysis of High Wavenumber Region

**Figure 2:**
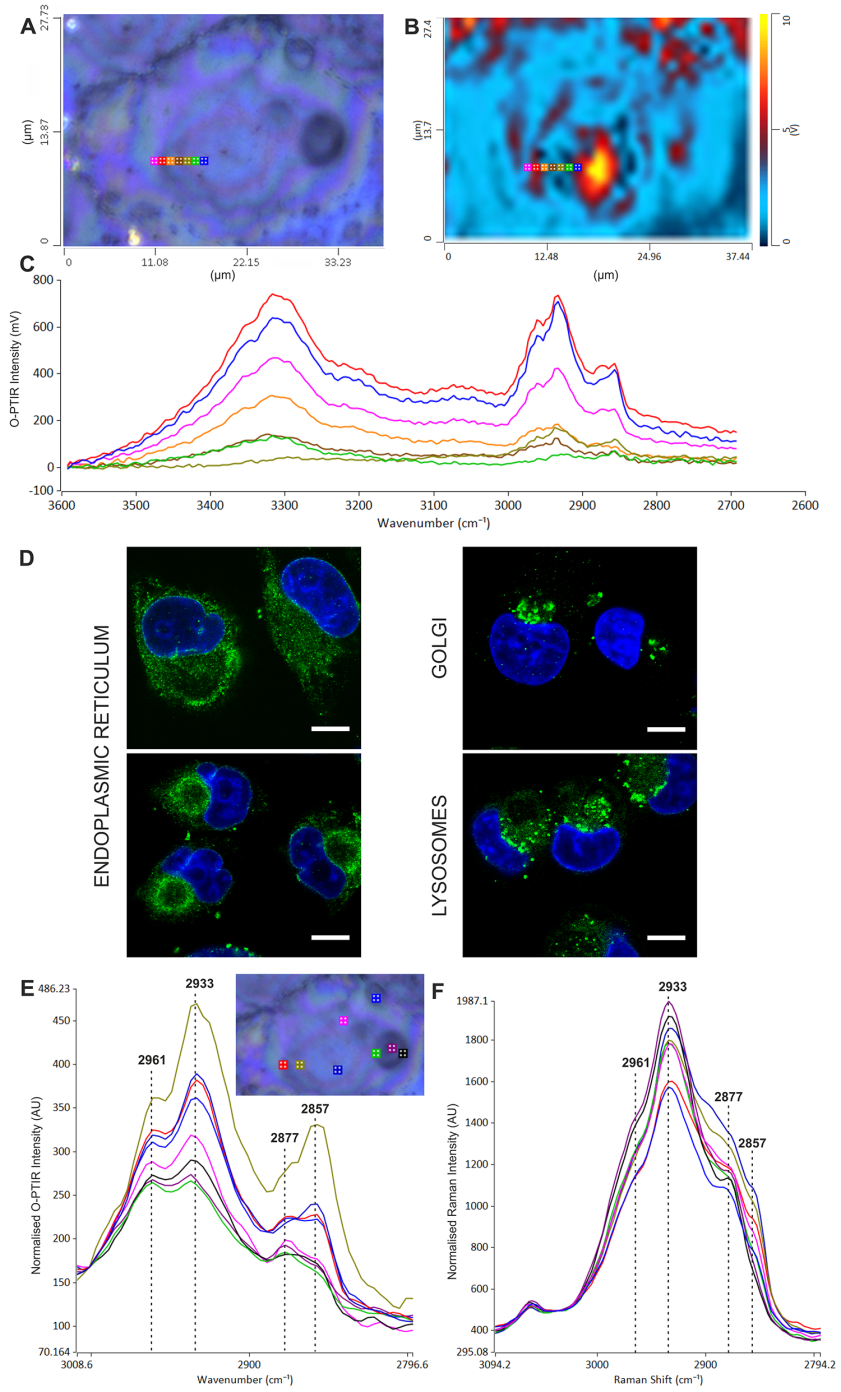
(A) Optical imaging of fixed unstained MIA PaCa-2 cells indicating the position of a line scan with a 1 μm step size. (B) Discrete frequency infrared image at 2935 cm^–1^. (C) O-PTIR spectra of the line scan in (A). (D) Confocal microscopy images of MIA PaCa-2 cells showing nuclei in blue (all panels), ER (left), Golgi apparatus (top right), and lysosomes (bottom right) all in green. Scale bar = 10 μm. Bright-field data are shown in Figure S5. (E) Representative OPO O-PTIR spectra (normalized to ∼3000 cm^–1^) and (F) concomitant Raman spectra (normalized to ∼3040 cm^–1^) showing the main CH_2_ and CH_3_ stretching regions. The colors of the spectra in (E) and (F) match the colored locations indicated in the inset image (from Figure 1B).

The variations in intensity in the high wavenumber region are examined further in Figure 2A–C. Figure 2A shows a further expanded optical image of the same MIA PaCa-2 cell of Figure 1, indicating the position of a line scan of spectra taken at 1 μm intervals. Figure 2B shows the discrete frequency image at 2935 cm^–1^ of the same area. The spectra from the line scan are presented in Figure 2C. The blue and the red spectra show high intensity in both lipid and protein, but there is significant variation in the lower intensity spectra. The green spectrum shows protein but virtually no lipid, whereas the olive green spectrum shows substantial C–H stretching band intensity, between ∼3000 and ∼2800 cm^–1^, but low amide A, suggesting very little protein and a highly localized area of mainly lipid. This could be a lipid droplet or an area associated with the smooth ER membrane, which consists of lipid (in the absence of ribosomes) and synthesizes cholesterol and phospholipids. This is consistent with our confocal images (Figure 2D) of MIA PaCa-2 cells and literature evidence. In the nucleus, in some cases, darker circular areas are identified^69^ that are consistent with the darker areas detected with the optical camera (panel A). The endoplasmic reticulum (ER) can appear in either a tubular shape or circular. It has been reported that the ER generally found in mammalian cells is typically in a tubular shape, but certain cells become more rounded during mitosis making the ER shape and therefore location difficult to interpret.^70^ The Golgi apparatus appears as a group of vesicles next to the nucleus and the lysosomes as spherical bodies across the cytoplasm. The C–H stretching region shows notable changes within the overall envelope, suggesting spatial variation mainly in the lipid distribution. Panels E and F in Figure 2 show O-PTIR and Raman spectra covering the C–H stretching region from locations within the cell depicted in Figure 1B. Note that the spectra have been normalized to take into account the different thicknesses of the cell at each location. Principally, four bands are observed associated with CH_2_ and CH_3_ groups, namely, the ν_as_CH_3_ at 2961 cm^–1^, ν_as_CH_2_ at 2933 cm^–1^, ν_s_CH_3_ at 2877 cm^–1^, and ν_s_CH_2_ at 2857 cm^–1^. It is interesting to note that there is generally more variation in total intensity in the O-PTIR spectra in Figure 2E compared with the Raman spectra in Figure 2F. In the O-PTIR spectra, in the region of the nucleolus (green, purple, and black spectra), both the CH_2_ bands (3933 and 2857 cm^–1^) drop significantly in intensity with respect to the CH_3_ bands (2961 and 2877 cm^–1^). This is observed by the complete switch in the ratio of the 2877/2857 cm^–1^ bands on moving from the nucleolus to elsewhere in the cell, indicating a lack of long-chain lipids in the nuclear region. This switch is also seen in the Raman spectra although the 2857 cm^–1^ band is not so pronounced compared with that observed with the O-PTIR.

#### Analysis of Amide I Region

In infrared spectroscopy of biological systems, the amide I region of the spectrum is of great interest since it has the potential to provide information regarding the secondary structure of proteins.^68^ Spatially resolved IR spectra of cells, using traditional FTIR, are, however, difficult to obtain for a number of reasons. First, the observed spectrum is the average of all of the proteins in a large area (several microns) and, second, the Mie scattering of infrared radiation from cells is most problematic in the region of the strongest vibration, namely, the amide I band, which exhibits the largest change in the real part of the refractive index *n* and hence caution must still be used in any assignment even after scatter correction.^40,41,44^

**Figure 3:**
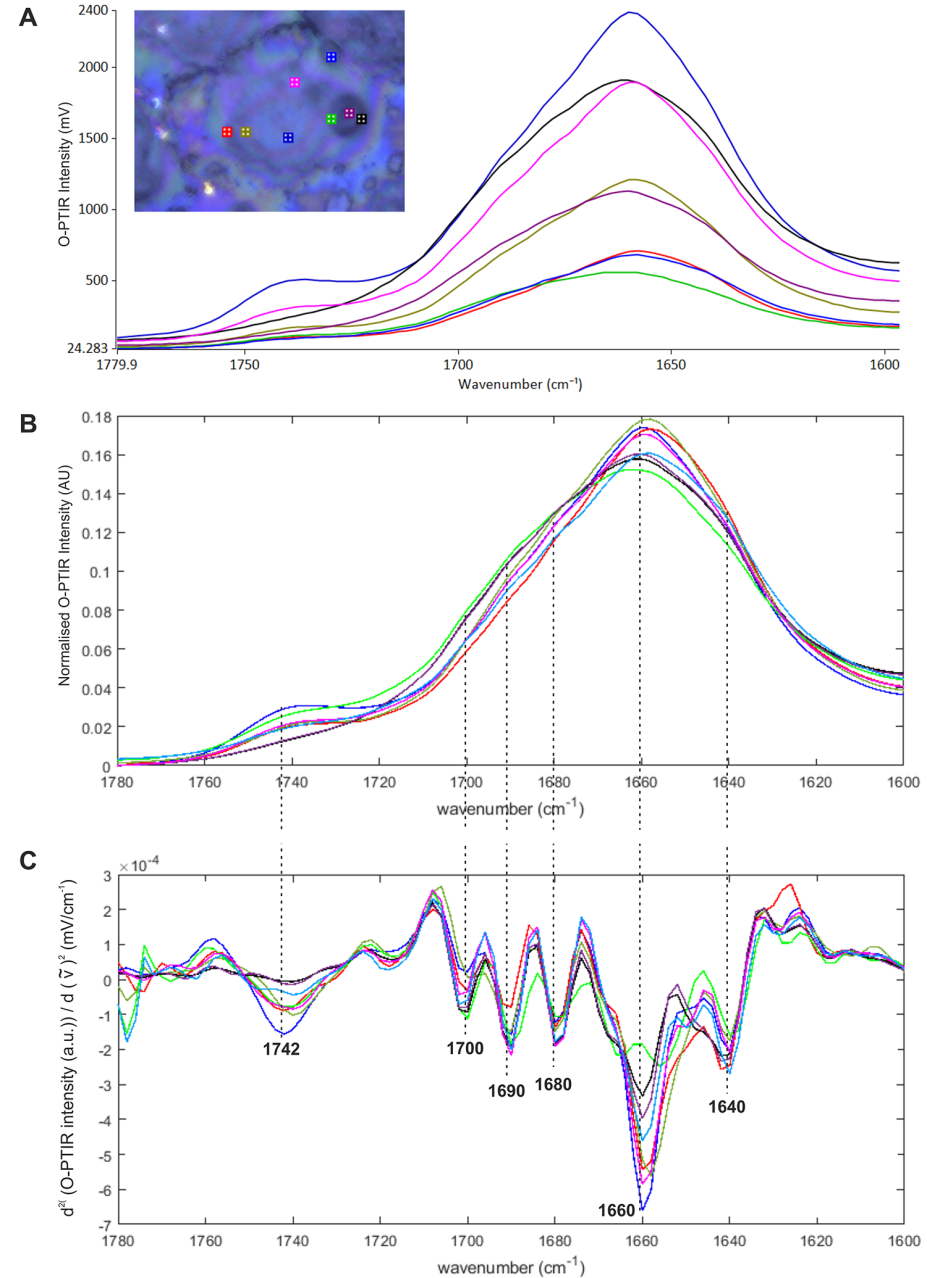
(A) QCL O-PTIR highlighting the amide I band with the colors matching the locations of the cell shown in the inset (top left). (B) The same spectra vector-normalized (MatLab). (C) Second derivative (MatLab).

Figure 3A shows O-PTIR spectra of the amide I band and the carbonyl region of the spectrum. As can be seen, there is considerable variation in intensity, which broadly, but not completely, relates to different thicknesses of the cell at that particular location. It is noteworthy that there is no evidence of Mie scattering or other scatter artifacts. Changes in the band position and shape are observed, and these are best seen in the vector-normalized spectra in Figure 3B and the second derivative of these spectra in Figure 3C.

The most interesting features are the changes associated with the spectra from the nucleolus (green, purple, and black spectra). The carbonyl band at 1742 cm^–1^ is almost completely absent in the nucleolus consistent with the lack of phospholipids, confirmed by the reduction of the CH_2_ vibrations in Figure 2E. The amide I vibration is predominantly (∼80%) a C=O stretch and is therefore strongly influenced by hydrogen bonding between adjacent peptide chains. The overall amide I band shape and position, therefore, are strongly affected by the secondary structure of the proteins.^68^ The second derivative of the amide I band, Figure 3C, shows this to great effect. The bands around 1620 and 1640 cm^–1^ are assigned to β-sheet, the bands around 1690 and 1617 cm^–1^ to antiparallel β-sheet, and the bands at 1656 cm^–1^ to α-helix, while the bands at 1676 cm^–1^ are assigned to either β-sheet or turns and bends. A strong difference in the protein content was observed in the nucleolus, and this was highly dependent on where the spectra are taken from. The amide I feature in the green, purple, and black spectra has very different intensities in Figure 3A but quite similar spectral band shapes when normalized in Figure 3B. Inside the nucleolus, there is a general shift in the amide I band to higher frequency accompanied by broadening of the band. The histone proteins in the nucleus are largely α-helix in structure, so the broadening is unlikely to be an increase in β-sheet. We therefore attribute this to the increase in DNA (1665–1660 cm^–1^) and RNA (1690 cm^–1^) in the nucleolus (black spectrum).

#### Raman Spectra of Low Wavenumber Region

**Figure 4:**
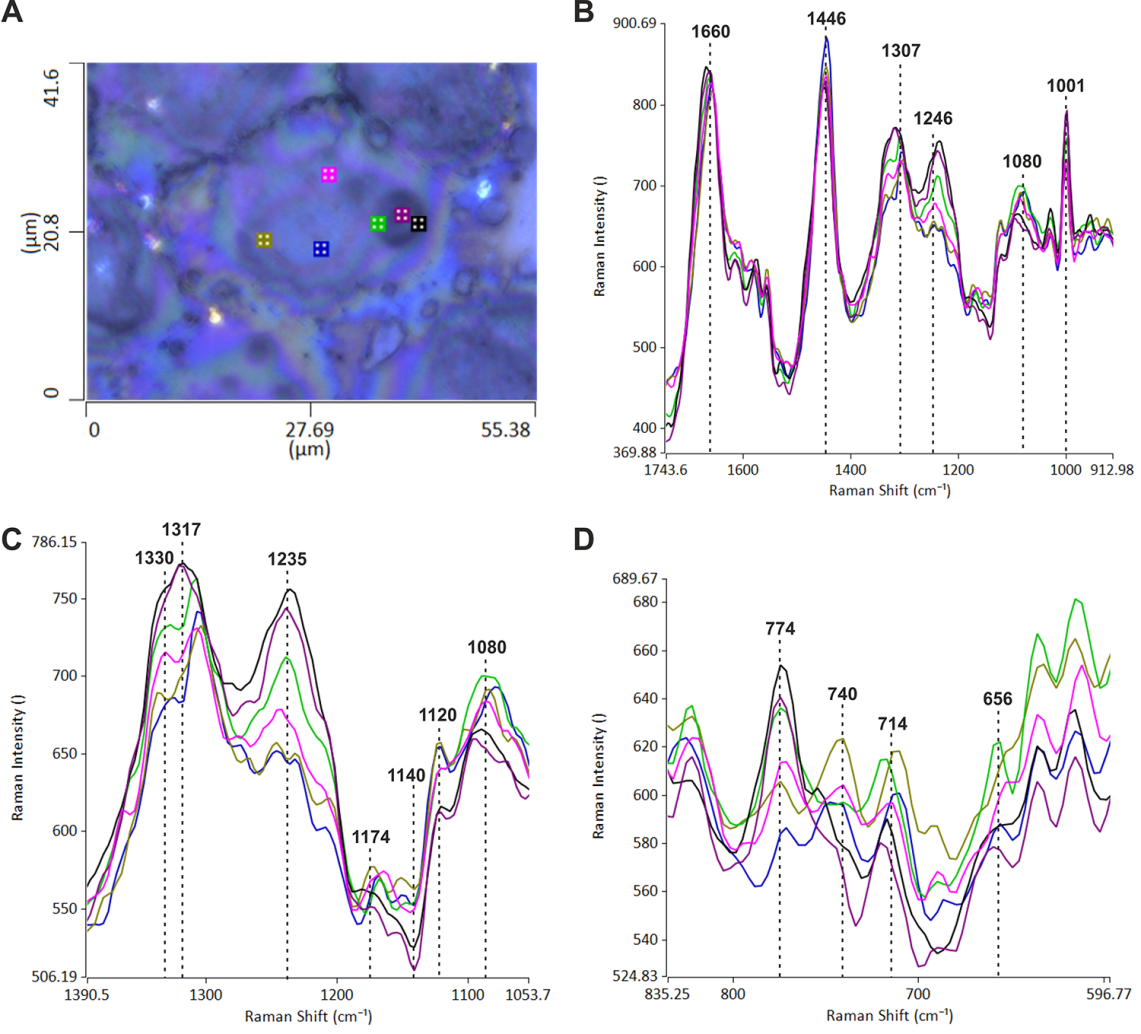
(A) Optical image of MIA PaCa-2 cell. (B) Raman spectra of the range showing the most intense features in the spectrum. (C) Expanded region between ∼1390 and ∼1053 cm^–1^ and (D) the region between ∼835 and 596 cm^–1^.

Figure 4A shows the optical image indicating the location of the Raman spectra (collected simultaneously with the O-PTIR data in Figure 3), and Figure 4B shows the Raman spectra from 1743 to 912 cm^–1^. The amide I at 1660 cm^–1^ is still reasonably strong, but the amide II is much weaker compared with the O-PTIR spectrum. The strongest band in the region is the 1446 cm^–1^ due to δCH_2_. Note that this is higher in relative intensity but slightly lower in frequency than observed in the O-PTIR. The cluster of bands between 1340 and 1240 cm^–1^ in Figure 4C represents the amide III vibrations and consists of combinations of N–H and C–H deformations.^71^ The 1237 cm^–1^ is assigned to the symmetric PO_2_^–^ stretch, which is mainly from DNA and RNA and, as can be seen, it is significantly higher in the nucleolus compared with elsewhere.^72^ The region from 1240 cm^–1^ to the phenyl aniline peak at 1001 cm^–1^ is complex and so the assignment of individual bands is problematic. However, this region also shows the sharp differences dependent on location. The purple spectrum in the center of the nucleolus shows a significant loss of intensity in this region, yet the green spectrum on the edge of the nucleolus (3 μm apart) has more intensity at 1140 cm^–1^ and the olive green spectrum at the edge of the ER has an increase at 1174 cm^–1^. Figure 4D shows the expanded region from 835 to 596 cm^–1^. The most interesting features are the triplet of bands at ∼774, ∼740, and ∼714 cm^–1^, which are found in both DNA and proteins.^73^

In DNA, the band at 774 cm^–1^ is much stronger than the other two with the 744 cm^–1^ being the weakest. In proteins, however, the 774 cm^–1^ band completely disappears, but the central band increases. The intensity of the lower frequency band remains similar in both, but there is a shift to lower wavenumber in the protein spectrum. These changes are exactly as those observed here on moving from the nucleolus (black spectrum) to the ER (olive green).

#### O-PTIR and Concomitant Raman for Live Cell Analysis

##### Overview

Having established that subcellular spatial resolution images can be obtained from fixed cells, data was collected from live cells in aqueous media. Live cells in PBS buffer were analyzed for two different cell lines, after being contained in the sandwich assembly, as indicated in Figure S2. Cell viability was confirmed by trypan blue staining, which can be seen in Figure S6.

**Figure 5:**
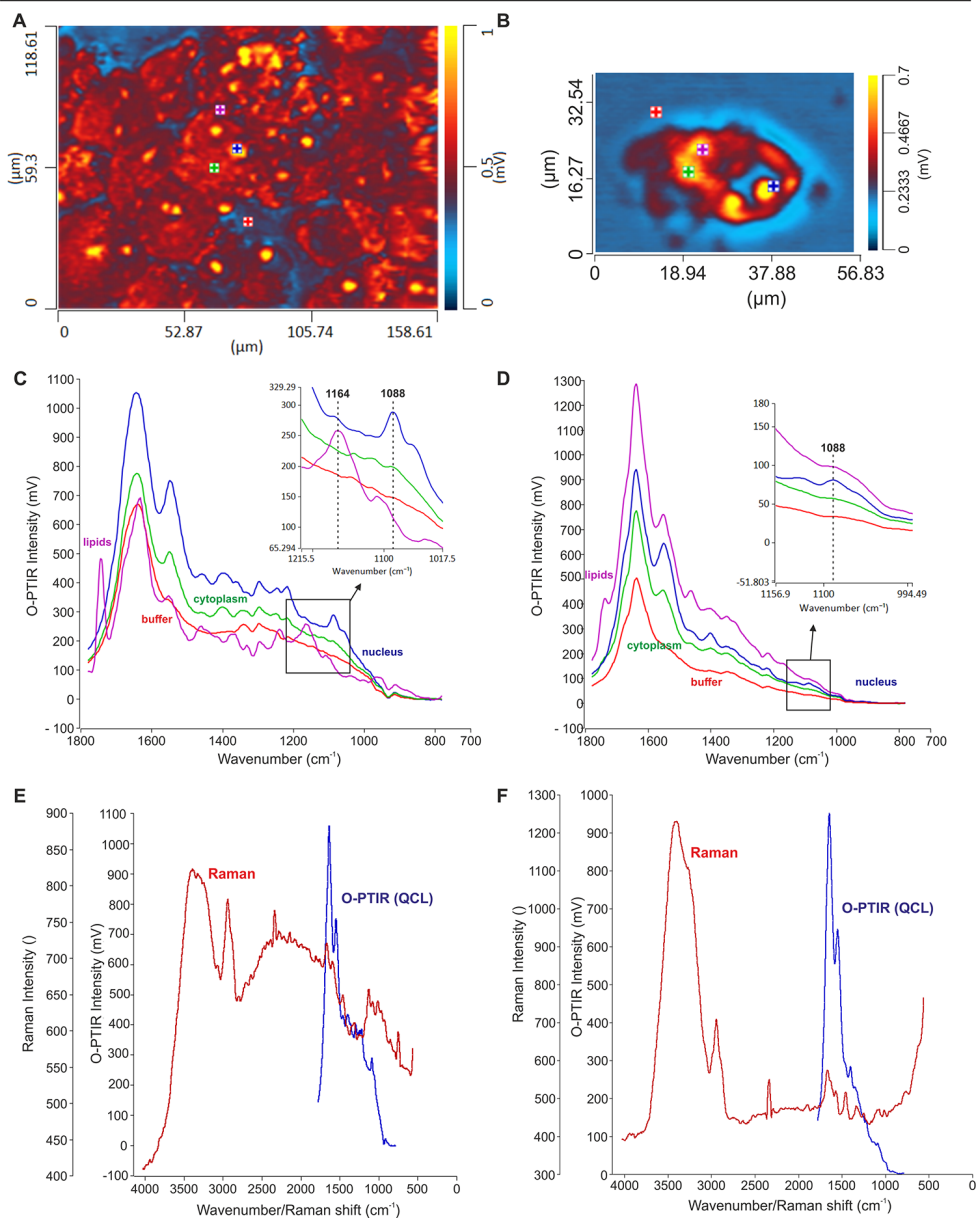
(A) Representative single-frequency images obtained at 1550 cm^–1^ for MIA PaCa-2 and (B) MDA-MB-231 live cells in PBS buffer. (C) O-PTIR spectra of different cell compartments as indicated by the colored points in the IR image in MIA PaCa-2 and (D) MDA-MB-23 live cells in PBS buffer. (E) Simultaneous IR (blue) and Raman (red) spectra obtained from MIA PaCa-2 and (F) MDA-MB-231 live cells in PBS buffer from the cellular compartments highlighted with blue points.

Initially, single wavenumber O-PTIR images were taken at 1550 cm^–1^ (amide II) in MIA PaCa-2 (Figure 5A) and MDA-MB-231 cells (Figure 5B) to identify the cell shape and structure (Figure 5C,D) and then IR (blue) and Raman (red) spectra were acquired (Figure 5E,F). The amide II spectral image was chosen because it is clearly distinct from the bending mode of water at approximately 1640 cm^–1^. Figure 5C,D shows examples of simultaneous QCL O-PTIR and Raman spectra obtained from an intracellular compartment in both cell lines. The water contribution in Raman is evident (from ∼3100 to ∼4000 cm^–1^), but it does not affect the biologically relevant peaks (below 3100 cm^–1^). In the O-PTIR spectra, the water contribution is minimal; in fact, the amide peaks appear completely resolved (∼1650 cm^–1^ amide I and ∼1550 cm^–1^ amide II) with an intensity ratio similar to that found in dehydrated cells. An explanation for this is that with the inverse sampling geometry being used (Figure S2), the infrared beam passes through the cell, which is adhered to the CaF_2_ surface before it passes through the water layer.

Figure 5C,D shows the QCL O-PTIR spectra obtained from various locations, both within and outside of cells. In the MIA PaCa-2 cells (Figure 5C), a peak at ∼1080 cm^–1^ (blue spectrum) appeared when the spectrum was acquired in a high-intensity spot (blue point in the O-PTIR image), a structure that we hypothesize as being the nucleolus. This is supported by the fact that the 1080 cm^–1^ band is assigned predominantly to the PO_2_^–^ symmetric stretch of phosphodiester groups in nucleic acids. The increased prominence of this feature, compared with the fixed cell spectra, is consistent with the change in DNA conformation from A-DNA (dehydrated) to B-DNA (hydrated) forms.^72^ Spectra acquired outside this region (green spectra, Figure 5C) show a significantly diminished 1080 cm^–1^ peak, which is indicative of the lack of nuclear material consistent with the cytoplasm. A peak at ∼1080 cm^–1^ was also observed in MDA-MB-231 cells (Figure 5D), although slightly less prominent compared to MIA PaCa-2 cells. Both cell lines have regions with strong C=O lipid signal at ∼1740 cm^–1^ (purple spectra, Figure 5C,D). Spectra were acquired from outside the cells (red spectra, Figure 5C,D) to observe the absorbance of the buffer: a strong peak at ∼1650 cm^–1^ was detected due to water absorption, while other biologically relevant peaks were lost.

##### QCL O-PTIR Spectra Cell in Aqueous Media

**Figure 6:**
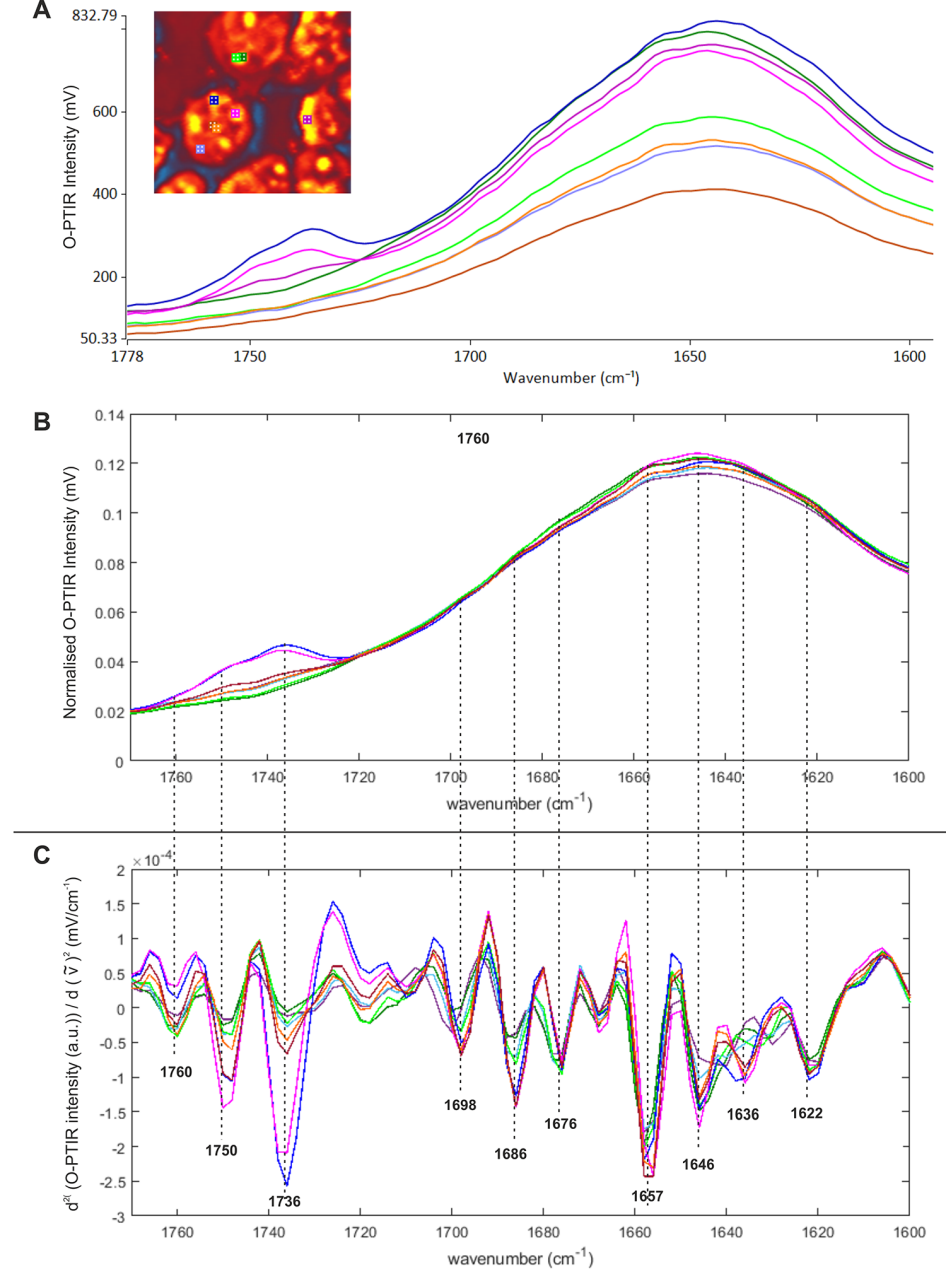
(A) Representative O-PTIR spectra of the amide I region of the spectrum, with the location of the spectra shown in the inset single-frequency image (top left) obtained at 1550 cm^–1^ in MIA PaCa-2 cells. (B) The same spectra vector-normalized (MatLab). (C) Second derivative (MatLab).

Figure 6 shows the representative O-PTIR spectra in the region of the amide I band spectrum, with the location of the spectra shown in the inset single-frequency image (top left) obtained at 1550 cm^–1^ from the MIA PaCa-2 cells in aqueous media. The amide I band and the carbonyl feature at ∼1740 cm^–1^ in Figure 6 are both clearly discernible despite the presence of the water. Most importantly, in the second derivative spectra (Figure 6C), there is a clear structure in the amide I band. Given that there is no structure in the liquid water spectrum, the observed spectral features are attributed to changes in the secondary protein structure at different locations within the cells. The main features observed in the second derivative spectra (Figure 6C) agree well with those in the dehydrated spectra (Figure 3C). However, the carbonyl band observed in the live cell spectra is split into two, with the main features at 1750 and 1736 cm^–1^. These are strongest in the blue, pink, and purple spectra, which are likely to be lipid droplets. However, note that these bands are completely absent in the green spectra, which are also obtained from areas of high intensity at 1550 cm^–1^. The main amide I feature is at 1646 and 1657 cm^–1^, attributed mainly to the α-helix proteins.

**Figure 7:**
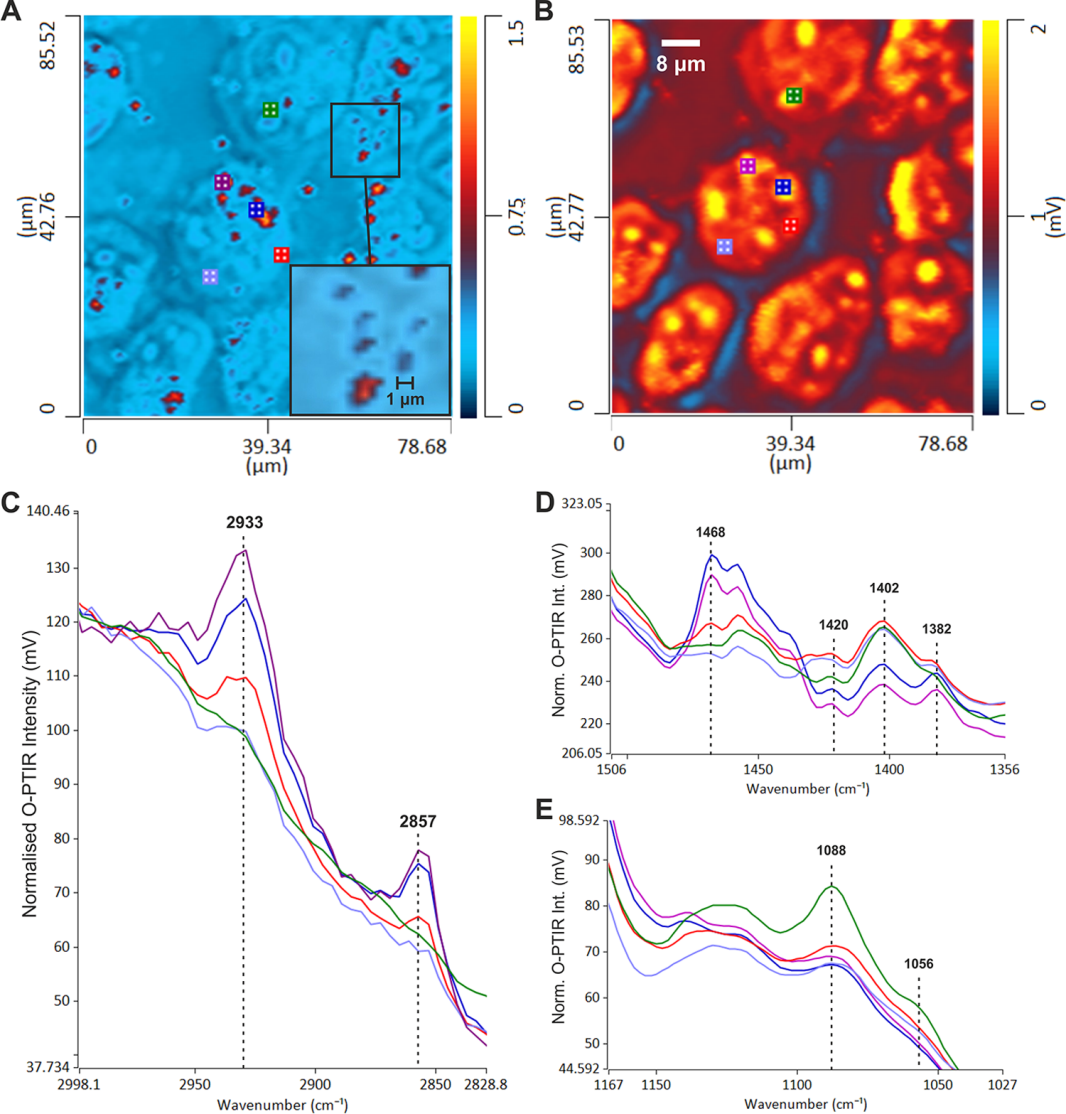
Representative single-frequency image of MIA PaCa-2 live cells in PBS buffer at (A) 2929 cm^–1^ and (B) 1550 cm^–1^. (C) Spectra obtained with OPO laser from locations indicated by the colored spots in the image in (A), highlighting the C–H stretching region. Spectra obtained with QCL laser from locations indicated by the colored spots in the image in (B), highlighting the regions 1506–1356 cm^–1^ (D) and 1167–1027 cm^–1^ (E).

Figure 7 shows MIA PaCa-2 cells analyzed with both OPO and QCL lasers. Figure 7A,B shows single-frequency images of the cells at 2929 and 1550 cm^–1^, respectively. Note that the bright spots highlighting the lipid-rich regions in Figure 7A do not coincide with the bright spots in Figure 7B, although for some there is a degree of overlap indicating that some areas are rich in both lipids and proteins, while others are not. The smallest features discernible in the lipid image are approximately <1 μm (see the inset in Figure 7A and an expanded version in Figure S4). Figure 7 also shows the expanded spectra in the lower frequency region. The regions 1506–1356 and 1167–1027 cm^–1^ are shown in Figure 7D,E, respectively. Here, we contrast the bright spots highlighted by the amide II band at 1550 cm^–1^ in Figure 7B, indicating high protein, but show that the areas have very different chemical compositions. The blue and pink spectra from the top region of the central cell in Figure 7B show strong features at 1468 and 1458 cm^–1^ indicative of relatively high lipid content. This is supported by the OPO data with the blue and pink spectra indicating high lipid content. In contrast, the green spectrum taken from a very similar looking bright spot on the lower part of the upper cell in Figure 7B shows very little signal at 1468 cm^–1^ but has more spectral features in common with the red spectrum, which is not associated with particularly bright features in the image at 1550 cm^–1^. In addition, the green spectrum shows strong intensity at 1088 cm^–1^ assigned to the symmetric PO_2_^–^ stretch of B-DNA and increased intensity at 1056 cm^–1^ assigned to the ν(C–O) in ribose RNA. It is clear, therefore, that although both areas are highlighted by the amide II band, one is almost certainly located in the nucleus where the other is likely to be a lipid-rich organelle.

## Discussion

The ability to obtain high spatial resolution (submicron) infrared spectra of biological cells across the majority of the mid-infrared range is a significant new capability, which will enable more detailed future studies of cells, drug–cell interactions, and cell responses to external stimuli. Obtaining the full infrared spectrum is important, and here, we have demonstrated the first use of the OPO-generated O-PTIR spectra for the study of single cells. In addition, in the system presented here, the instrument architecture enables the collection of both IR and the concomitant Raman spectra from the same spot with the same spatial resolution. This is possible because Raman excitation is inherent to the green probe laser.

To extract Raman spectral information, Raman-shifted photons are optically separated and sent to the Raman spectrometer, while the unshifted (Rayleigh-scattered) light passes through to the visible O-PTIR detector for signal processing and IR spectral extraction.^74^ The main advantage of this arrangement is the step change in spatial resolution of the IR hyperspectral imaging due to the shortened wavelength of the visible probe beam. In addition, unlike conventional IR microscopy, where resolution is strongly wavelength-dependent, the O-PTIR spatial resolution is constant along the entire spectrum. This gives a ∼30× improvement in spatial resolution at the longer wavelengths (see Figure S3).

While a certain degree of pixel oversampling is required to ensure diffraction-limited performance is attained,^75,76^ oversampling by more than a factor of ∼3 will not improve spatial resolution and is generally termed “empty magnification”.^77,78^ Therefore, despite a pixel size of ∼1.1 μm or less, in some commercial systems, this equates to an oversampling of ∼9 at longer wavelengths and will still result in a relatively poor spatial resolution of ∼10 μm. A more subtle and often overlooked advantage of O-PTIR is that the spectra collected from the just-resolved features in the sample are relatively pure across the entire spectrum, i.e., they will have little to no “spatial blur” from the surrounding areas of samples. Having well-resolved images, free from spatial blur, is of critical importance and something that O-PTIR uniquely delivers.

This unique advantage affords benefits in the investigation of biochemical changes in biological samples after exposure to certain agents (i.e., drugs, radiation, nanoparticles). Previous studies have focused on the comparison between IR and Raman data obtained from biological samples. Despite comparing multiple vibrational spectroscopy techniques, the different resolution and pixel size often make it impossible to obtain comparable chemical information from the same area of the sample by these methods.^79^ Here, we are able to:

(i) Identify organelles in fixed cells and live cells using both O-PTIR and Raman;
(ii) Observe an increase in β-sheets or RNA bands, consistent with the nucleolus, which is the site where new ribosomes are assembled; therefore, it contains rRNA (essential component of the ribosomes) and proteins;^69^
(iii) Identify lipid-rich regions assigned to the ER from consideration of their localization in the cell (compared to our confocal fluorescence microscopy data) and the OPO IR image;
(iv) Obtain, from the live cells in aqueous buffer, high-quality discrete frequency images (Figures 5A,B and 7A,B) despite the presence of the water layer, enabling the identification of lipid droplets ∼1 μm or less.

## Conclusions

In this paper, we have demonstrated the first use of OPO and QCL excitation O-PTIR and concomitant Raman for spectroscopic analysis of fixed and live biological cells. We show that it is possible to obtain IR and Raman spectra in the diagnostically useful fingerprint region at a <1 μm spatial resolution. IR spectral data are collected in a noncontact (far-field) mode and are obtained without any dispersive scatter artifact (such as the Mie scatter) that can plague conventional FTIR/QCL microscopes, thus improving the confidence of spectral interpretations of subtle spectral differences. Most importantly, we demonstrate that we can identify an undistorted amide I band in live cells in an aqueous environment, which opens up the possibility of detecting subtle protein secondary structure changes, normally obscured by the presence of water. This is a significant breakthrough for the spectroscopic analysis of biological systems. The concomitant Raman spectra yield additional complementary information regarding the cells and demonstrate that this new combined methodology can provide enhanced spectroscopic analysis.

## Supporting Information Available

Typical band assignments for infrared spectra of mammalian cells; schematic diagram of the layout of the optical system; scheme of the sample preparation for live cell analysis; theoretical spatial resolution comparisons; example of 0.5 μm features; confocal microscopy images of MIA PaCa-2 cells; and cell viability (PDF)
