# Supplementary material for: Analysis of Fixed and Live Single Cells Using Optical Photothermal Infrared with Concomitant Raman Spectroscopy
Source: Anal Chem. 2021 Feb 17;93(8):3938–50. doi: 10.1021/acs.analchem.0c04846 (PMC8018697; doi:10.1021/acs.analchem.0c04846)
Supplement: Supplementary file 1 — ac0c04846_si_001.pdf [file ac0c04846_si_001.pdf]

Supporting Information

Analysis of fixed and live single cells using optical photothermal infrared with concomitant Raman spectroscopy

Alice Spadea[a,b], Joanna Denbigh[cd], M. Jayne Lawrence[a,b], Mustafa Kansiz[e] and Peter Gardner[f,g]*

a NorthWest Centre for Advanced Drug Delivery (NoWCADD) School of Health Sciences University of Manchester Oxford Road, Manchester M13 9PL, UK

b Division of Pharmacy and Optometry Faculty of Biology, Medicine and Health University of Manchester, Manchester Academic Health Science Centre Oxford Road, Manchester M13 9PL, UK

c Seda Pharmaceutical Development Services, Alderley Park, Alderley Edge, Cheshire SK10 4TG.

d Formerly: School of Science, Engineering and Environment, University of Salford, Salford, M5 4WT

e Photothermal Spectroscopy Corp. 325 Chapala Street, Santa Barbara, CA 93101, USA

f Manchester Institute of Biotechnology, University of Manchester, 131 Princess Street, Manchester, M1 7DN, UK

g Department of Chemical Engineering and Analytical Science, School of Engineering, University of Manchester, Oxford Road, M13 9PL, UK

Table of contents: **Page**

Table S1. Typical band assignment for infrared spectra of mammalian cells S1
Figure S1. Schematic diagram of the layout of the optical system S2
Figure S2. Scheme of the sample preparation for live cell analysis S3
Figure S3. Comparison of theoretical IR spatial resolutions S3
Figure S4. Example of 0.5 µm features - single frequency image MIA PaCa-2 live cells S4
Figure S5. Confocal microscopy images of MIA PaCa-2 cells showing organelles S4
Figure S6. Viability of MIA PaCa-2 and MDA-MB-231 cells while in the "sandwich" S5
Methods for cell viability S6

Table S1. *Typical band assignments for infrared spectra of mammalian cells collated and amended from refs [1-6].*

Wavenumber /cm^{-1}	Band assignments of infrared spectra from mammalian cells<br>Functional group assignment	Biomolecule
3300	Amide A νN-H	Protein
3060	Amide B νN-H	Protein
2970-2950	ν_{as} CH_3	Lipid
2935-2915	ν_{as} CH_2	lipid
2880-2860	ν_s CH_3	lipid
2860-2850	ν_s CH_2	lipid
1742-1730	ν(C=O) carbonyl	Phospholipids
1717	ν(C=O) carbonyl	B DNA
1712	ν(C=O) carbonyl	A DNA
1695-1674	Amide I, νC=O (70-85%) and νC-N (10-20%) β-sheet	Protein
1690	RNA ν(C2=O)	RNA

1686-1662	Amide I, νC=O (70-85%) and νC-N (10-20%) turns and bends	Protein
1665-1660	DNA, ν(C_5=O), δ(N=H), RNA, ν(C_6=O)	DNA RNA
1650-1648	Amide I, νC=O (70-85%) and νC-N (10-20%) α-helix	Protein
1641-1633	Amide I, νC=O (70-85%) and νC-N (10-20%) β-sheet	Protein
1610-1628	Amide I, νC=O (70-85%) and νC-N (10-20%) aggregated strands, antiparallel β-sheet	
1570-1530	Amide II, δN-H (40-60%), νC-N (18-40%) and νC-C (10%)	Protein
1465	δCH_2	Lipid
1452	$δ_{as}$ CH_3	Lipid
1397	ν-COO^-	lipid
1379	$δ_s$ CH_3	lipid
1340-1240	Amide III Coupled N-H/C-H deformations	Proteins
1240-1237	$ν_{as}$ PO_2^-	DNA RNA phospholipids
1150	νC-O and δC-O-H	Carbohydrates
1099-1080	$ν_s$ $PO2^-$	DNA RNA phospholipids
1063	$ν_s$ PO_2^- , νC-C, νC-N,	B-DNA
1050	$ν_{as}$ OPO	RNA, Phosphate esters

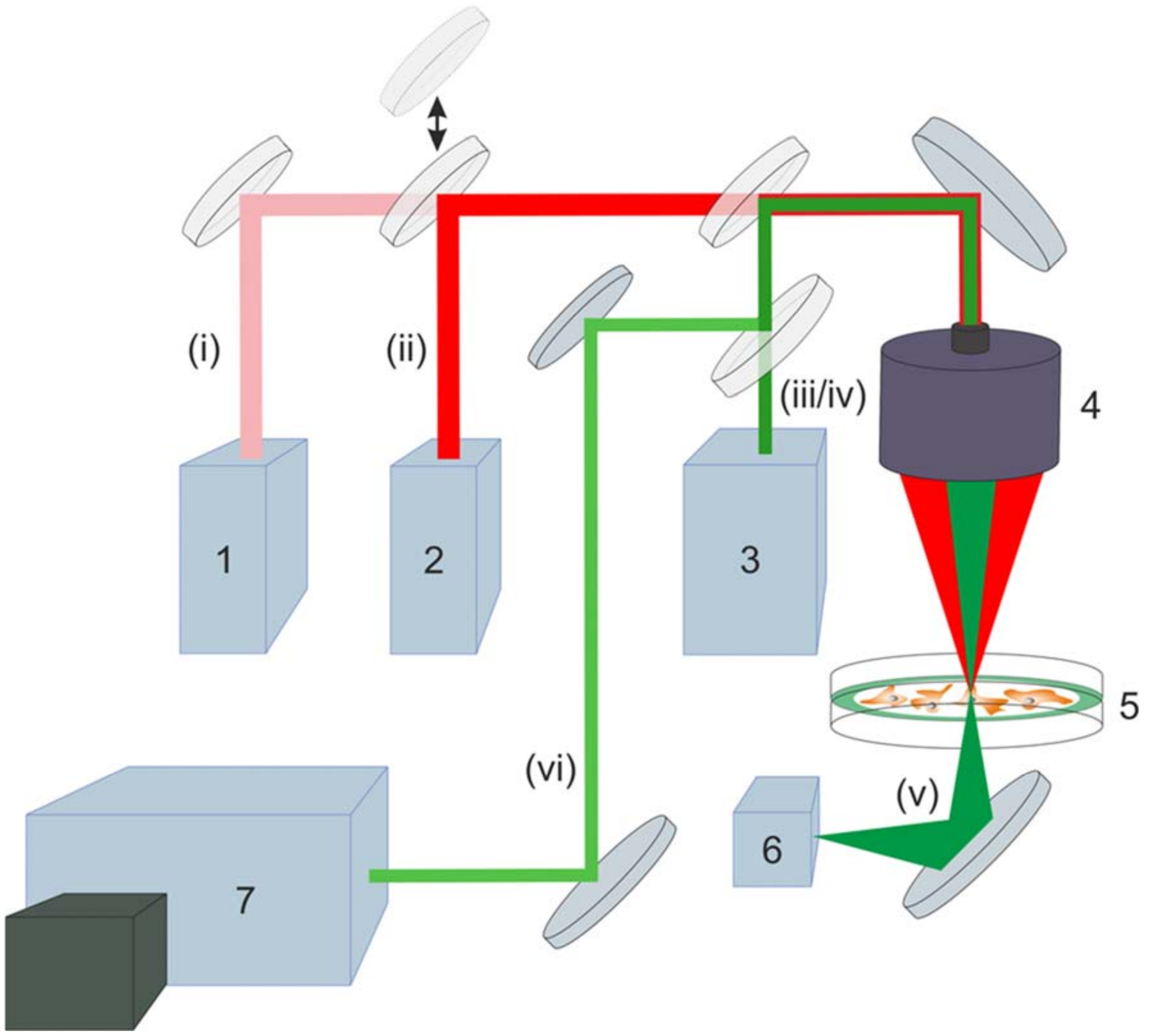

Figure S1. A schematic diagram of the layout of the optical system. (1). An OPO tuneable infrared laser (3600-2700 cm^{-1}) (2) A four chip tuneable QCL laser (1890-790cm^{-1}). (2) (3) A green (532 nm) probe laser and visible detector. (4) All reflective cassegrain microscope objective. (5) A sample holder containing live cells in an aqueous layer between two CaF_2 windows sealed with a hydrophobic seal. (6) visible detector and (7) Raman spectrometer. (i) OPO beam path (ii) QCL beam path (iii) back reflected probe beam (iv) Vis return (reflection mode) (v) Vis transmission mode probe beam (vi) Raman scattered beam.

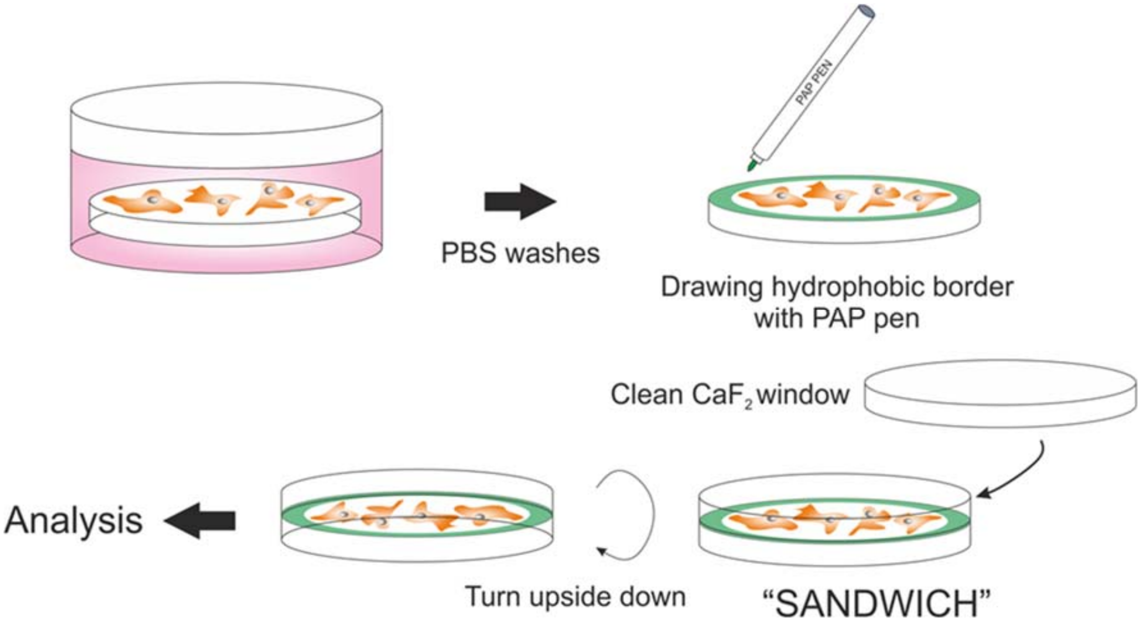

Figure S2. Scheme of the sample preparation for live cell analysis with Photothermal. Note that a thin layer of PBS is left on top of the cells prior to sandwich assembly therefore the cells remain hydrated during analysis.

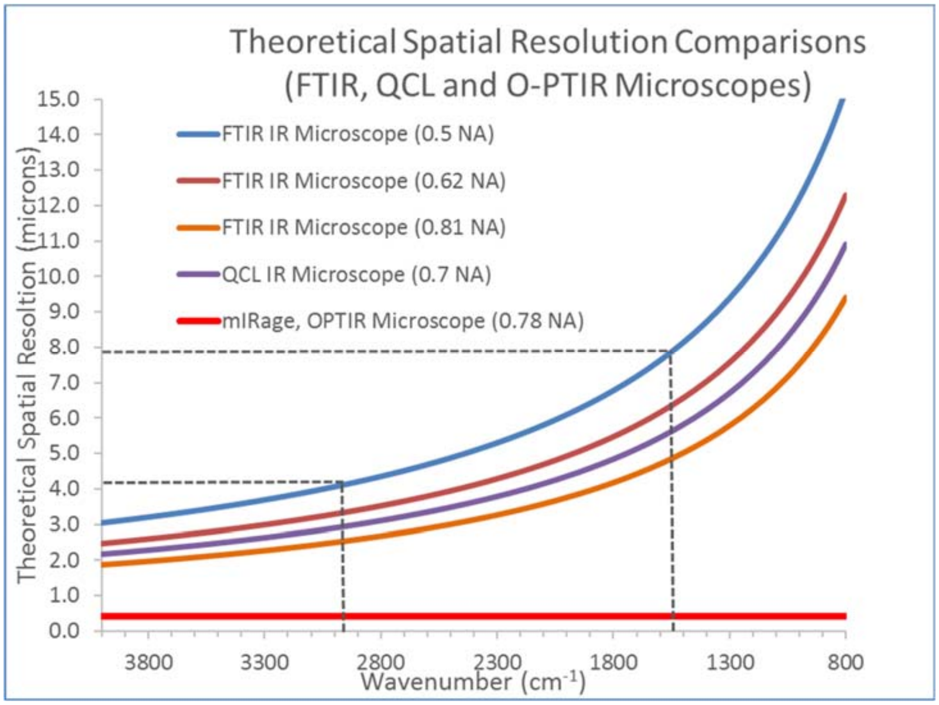

Figure S3. Comparison of theoretical spatial resolution for traditional IR microscopy (FTIR/QCL) and O-PTIR microscopy (adapted from Kansiz *et al*. [7]).

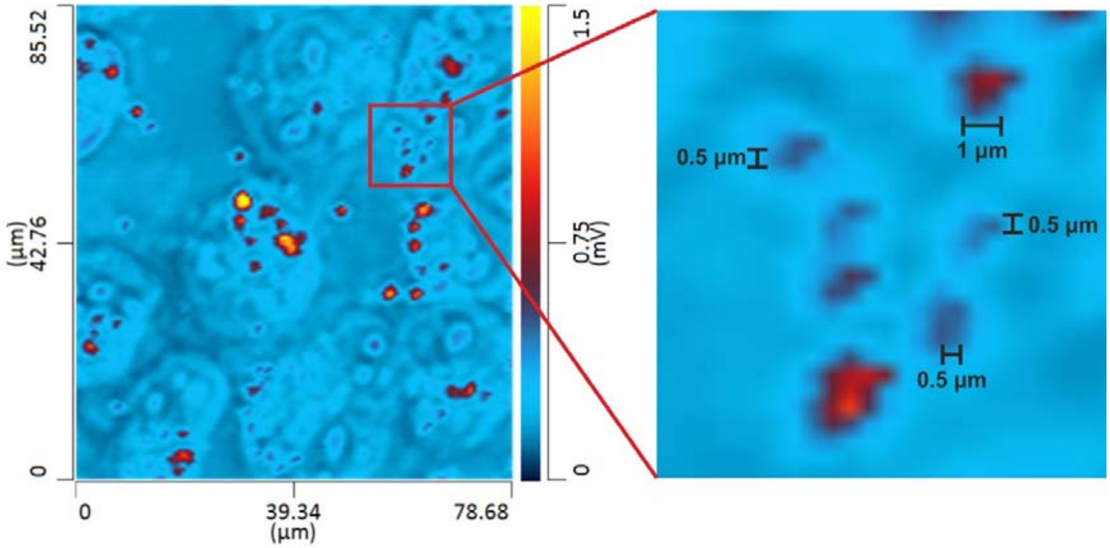

Figure S4. Example of 0.5 µm features obtained from a single frequency image of MIA PaCa-2 live cells in PBS buffer at 2929 cm^{-1} using the OPO laser.

Investigating cell structures with confocal microscopy

To investigate the localisation of organelles made of lipidic vesicles inside the cells, a qualitative screening was performed using laser scanning confocal microscopy. Figure S5 shows the main lipid organelles inside the cells, along with the brightfield images (bottom panels) to show the cell boundaries.

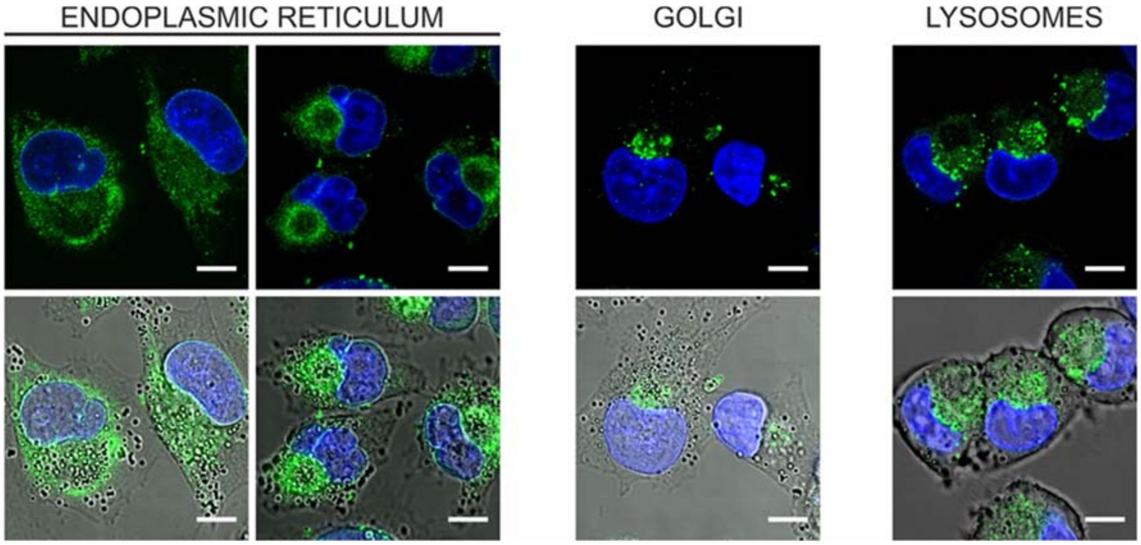

Figure S5. Confocal microscopy images of MIA PaCa-2 cells showing ER (left panals), Golgi apparatus (middle panels) and lysosomes (right panels). Top panels show nuclei (blue) and the different organelles (green). The bottom panels show the same images as above merged with the brightfield (grey) to identify cell borders.

Cell viability

The protocol we developed to analyze live cells in aqueous buffer requires the assembly of a "sandwich" with cells growing onto a CaF_2 window with another clean CaF_2 window placed on top, as explained in figure S2. Therefore, monitoring the viability of cells under measurement conditions was necessary. To ensure the reproducibility of the experiments, two cancer cell lines, MIA PaCa-2, pancreatic cancer, and MDA-MB-231, breast cancer, were tested. Cells were incubated with 0.2% (w/v) Trypan blue prior to "sandwich" assembly and the viability monitored up to 30 minutes, consistent with the longest analysis time for this experiment. Longer exposure times to trypan blue can also cause toxicity [8]. Figure S6 shows that cell mortality after 30 minutes in PBS in the "sandwich" is almost null in both cell line tested, as only very few cells turned blue during the trypan blue test.

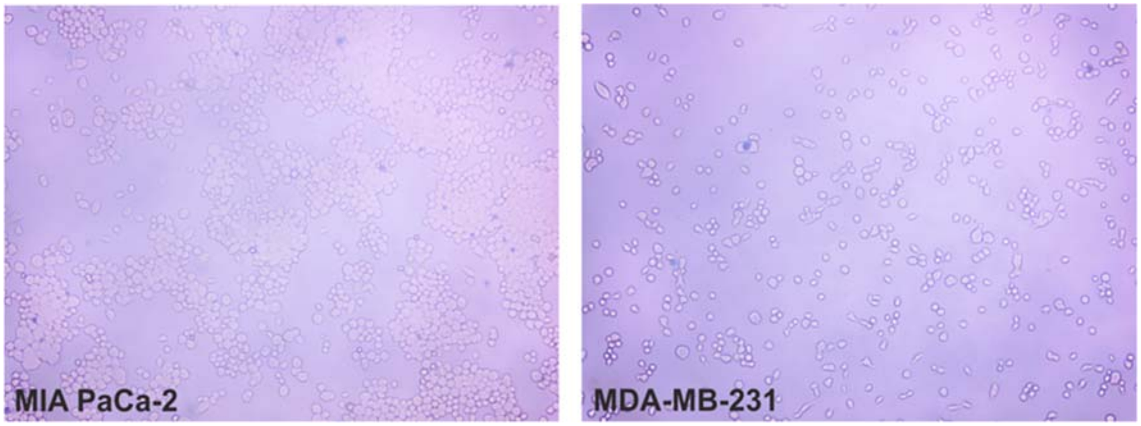

Figure S6. Sample images of respectively MIA PaCa-2 and MDA-MB-231 cells after 30 minutes incubation with 0.2% w/v trypan blue for viability assay. Images were taken with an optical microscope, 4X magnification objective.

METHODS SUPPORTING INFORMATION

Viability test

To monitor viability of cells in the "sandwich" over time, a solution of 0.2% (w/v) trypan blue in PBS was added to the CaF_2 window adherent cells. The same procedure for the "sandwich" preparation as described above was followed and the cell viability monitored up to 30 minutes. For this work, we did not monitor viability for longer periods of time since this is the maximum time cells were used for analysis with the instrument, and because incubation for long time periods with trypan blue could cause cell toxicity.

Supporting information references

[1] de Carvalho, A.L.M.B., et al., Chemotherapeutic response to cisplatin-like drugs in human breast cancer cells probed by vibra-tional microspectroscopy. Faraday Discuss, 2016. 187: p. 273-298.

[2] Legal, J., M. Manfait, and T. Theophanides, Applications of FTIR spectroscopy in structural studies of cells and bacteria. Journal of molecular structure, 1991. 242: p. 397-407.

[3] Matthäus, C., et al., Infrared and Raman microscopy in cell biology. Methods in cell biology, 2008. 89: p. 275-308.

[4] Zscherp, C. and A. Barth, What vibrations tell us about proteins. Q. Rev. Biophys, 2001. 35: p. 369-430.

[5] Movasaghi, Z., S. Rehman, and D.I. ur Rehman, Fourier transform infrared (FTIR) spectroscopy of biological tissues. Ap-plied Spectroscopy Reviews, 2008. 43(2): p. 134-179.

[6] Wood, B.R., The importance of hydration and DNA con-formation in interpreting infrared spectra of cells and tissues. Chem-ical Society Reviews, 2016. 45(7): p. 1980-1998.

[7] M. Kansiz, C. Prater, E. Dillon, M. Lo, J. Anderson, C. Marcott, A. Demissie, Y. Chen and G. Kunkel, Microscopy Today, 2020, 28, 3, 26-36.

[8] D. Awad, I. Schrader, M. Bartok, A. Mohr and D. Gabel, Comparative toxicology of trypan blue, brilliant blue g, and their combination together with polyethylene glycol on human pigment epithelial cells, Investigative ophthalmology & visual science 52 (2011), no. 7, 4085-4090.
